# Exploring the disease experience and supportive care for people with very severe chronic obstructive pulmonary disease in Malaysia: a multiperspective qualitative study

**DOI:** 10.7189/jogh.15.04127

**Published:** 2025-05-23

**Authors:** Zee Nee Lim, Su May Liew, Ee Ming Khoo, Hilary Pinnock, Sylvia McCarthy, Jayakayatri Jeevajothi Nathan, Yong Kek Pang, Nik Sherina Hanafi, Norita Hussein, Ahmad Ihsan Abu Bakar, Yun Li Chan, Aziz Sheikh, RESPIRE Collaboration

**Affiliations:** 1 Hospis Malaysia, Kuala Lumpur, Malaysia; 2 Department of Primary Care Medicine, Faculty of Medicine, Universiti Malaya, Kuala Lumpur, Malaysia; 3 Usher Institute, University of Edinburgh, Edinburgh, UK; 4 Department of Medicine, Faculty of Medicine, Universiti Malaya, Kuala Lumpur, Malaysia; 5 Hospital Pusrawi, Kuala Lumpur, Malaysia

## Abstract

**Background:**

In Malaysia, palliative services are almost non-existent for those with severe organ failure such as chronic obstructive pulmonary disease (COPD). Access to palliative care services and awareness among the public and healthcare professionals remain low. While recognised as important in high income settings, the literature on palliative care for non-malignant disease is relatively uncommon in low- and middle-income countries, and none considered the multicultural setting of Malaysia. We aimed to explore the views and experiences of patients, healthcare providers (HCPs) and policymakers about their experience of very severe COPD and palliative care.

**Methods:**

We undertook in-depth qualitative interviews with patients with very severe COPD, HCPs working in respiratory, palliative care, and primary care medicine, and health policymakers in Kuala Lumpur, Malaysia. Interviews followed a topic guide, were audio-recorded, transcribed verbatim, and analysed using a thematic analysis approach.

**Results:**

We conducted 22 in-depth interviews with patients (n = 11), and physicians working in respiratory (n = 4), palliative care (n = 4) and primary care (n = 3) medicine. Four main themes emerged. First, there was poor understanding regarding severe, potentially life-limiting COPD and the need for palliative care. Second, patients were suffering from the severe physical, emotional, and psychosocial impact of the disease. Third, there was a lack of accessible, compassionate, holistic, and coordinated care. Finally, cultural issues such as religious norms, spirituality, community, and power hierarchies influenced patient care and acceptance of their condition.

**Conclusions:**

The Malaysian healthcare system is responding poorly to the needs of patients with very severe COPD. Raising awareness of these needs is the first step, but there needs to be a major change within the system if the care of this hidden neglected population is to be improved.

Chronic obstructive pulmonary disease (COPD) is the third leading cause of death globally [1]. While medical treatments can reduce the symptom burden of this illness and quitting smoking can reduce progression, decline is inevitable and premature death is common [2]. Supportive and palliative care for those with severe life-threatening diseases to alleviate suffering is recognised to be a basic human right. Yet globally, many patients die without receiving such care [3]. Gore and colleagues [2] showed that patients with severe COPD had significantly worse quality of life than patients with lung cancer and none had received specialist palliative care. The ‘transition’ to end-of-life care is unclear for patients with COPD who experience a very prolonged ‘chaotic’ disease journey without the ‘public story’ of cancer patients. One effect of this long-trajectory but inexorable decline is that patients and their families adapt to the substantial and increasing disability. Needs are often not expressed and help is not requested [4].

Although the need for supportive end-of-life non-cancer care has received attention in high-income countries [5], this is not the case in low- and middle-income countries (LMICs). Although they bear the highest burden of NCDs, research from these contexts is severely limited [6]. In Malaysia, an estimated 100 000 patients per year would benefit from palliative care [7], but currently only 10% have access to services, 90% of whom are cancer patients [8]. Research is required to raise awareness; direct policy making and strengthen health systems. Malaysian healthcare services face the challenge of reconfiguring their services to meet the needs of the increasing number of people in the terminal phases of organ failure – and specifically, COPD [9]. It is unclear, however, whether this care should be the remit of specialist palliative care services, primary care services, or specialist pulmonologists. A key characteristic of Malaysian society is that it is multicultural; here we addressed this important gap in the existing body of knowledge by examining the unique interplay of cultural, structural, and systemic factors influencing COPD care in Malaysia.

We aimed to explore the views and healthcare experiences of the relevant stakeholders – namely patients with very severe COPD, physicians, and policymakers – regarding the disease experience and provision (or lack thereof) of supportive and palliative care for these patients and to inform the development of culturally-tailored, equitable, and actionable strategies to improve palliative care services for this underserved group.

## METHODS

We conducted this qualitative study over 18 months in 2019–20. The interviews were conducted based on this topic guide [10] (Appendix A in the **Online Supplementary Document**), which helped keep them structured.

### Recruitment

#### Patient participants' eligibility criteria

We asked respiratory, palliative, and primary care physicians to identify people with COPD with poor prognosis in need of supportive and palliative care using:

1. Global Initiative for Obstructive Lung Diseases (GOLD) severity staging that is very severe airflow limitation (forced expiratory volume (FEV_1_) less than 30% predicted) [11];
2. Supportive and Palliative Care Indicators (SPICT) Tool [12];
3. The surprise question ‘Would you be surprised if your patient were to die in the next 12 months?’ [13]. This question is used as part of the UK Gold Standard Framework that supports early identification of patients nearing the end of life [14]. Although the accuracy of the surprise question as a prognostic tool varies widely [15], it is still useful in this study as patients who answered ‘No’ are likely to need palliative care services.

The exclusion criteria were the inability to participate (for example, impaired cognition due to dementia) or the presence of concomitant life-threatening illness (for example, lung cancer which would have influenced their access to palliative care services). Patients identified by their treating physicians were assessed by clinical academics on the research team. There were no exclusions following referral. We purposively sampled participants based on age, sex, ethnicity, and recruitment setting to obtain a range of perspectives [16]. We gave participants at least three days to consider before taking their informed consent, after which we arranged one-to-one interviews at their home or other preferred venue. Where patients preferred to be interviewed in the presence of their carer, we noted down any relevant remarks made by the carer.

#### Eligibility criteria for healthcare provider participants

We recruited respiratory, palliative, and primary care healthcare providers (HCPs) in the Klang Valley, Malaysia regarding their views on this topic. We interviewed professionals who headed their unit and were policymakers in the hospitals, primary care division, palliative care division, and the Malaysian Ministry of Health. We arranged the interviews at the health service institution, workplace, or other convenient venue according to their preference.

We achieved data saturation [17] by the 10th interview for the patients and the ninth interview for the physicians. The interviews were conducted in the participant’s preferred language (English, Malay, Mandarin, or Tamil).

### Data collection

Experienced qualitative researchers (SML, SAM, ZNL) conducted the interviews, which lasted for up to an hour and audio-recorded them with the participants’ consent. YLC took field notes during the interview. Audio recordings were transcribed verbatim by a transcriber who removed identifiers and used study codes to preserve anonymity. Transcripts in Malay, Mandarin or Tamil were then translated into English for coding and analysis. Research assistants who were fluent in the languages used during the interview carried out the translation and transcribing which was then checked by the researcher who conducted the interview. No repeat interviews were conducted.

We adapted an interview guide from similar work [18] and developed it further for use with a list of open-ended questions covering broad themes (Appendices A and B in the **Online Supplementary Document**). We asked patient participants about their experience of living with COPD, their main physical, psychological, social, and spiritual concerns, views on the care and treatment they received from the healthcare system and society, and experiences of interactions with healthcare practitioners and the community. Issues covered with the physicians and policymakers included their experiences of looking after people with very severe COPD and their concerns (if any) with regard to current resource management [18] (Appendix B in the **Online Supplementary Document**). We also sought their views regarding barriers to care provision, and how services could be improved.

### Data analysis

We organised, managed, and coded the interview data using NVivo, version 10 (QSR International, Burlington, Massachusetts, USA) and analysed it using thematic approach, guided by established qualitative frameworks [19,20]. Researchers working in pairs (SML and YLC; SAM and CYL; ZNL and YLC) independently performed the coding, resolving discrepancies by discussion. They generated the initial codes inductively, capturing key features of the data relevant to the research questions. They then organised them into broader categories and developed overarching themes collaboratively through iterative discussions. Afterwards, they created a written and graphical summary of the issues for each code using the One Sheet of Paper method [21], visually mapping the relationships between codes and themes, thus aiding in the refinement of themes and sub-themes. The multidisciplinary research team held regular review sessions to evaluate the coding framework and emerging themes to address potential biases in data interpretation.

We integrated reflexivity into the process, with researchers maintaining reflective journals to document their assumptions, potential biases, and the rationale behind analytical decisions. Finally, we shared the results with participants for member-checking to validate the findings and ensure that the interpretations accurately reflected their views.

#### Reflexivity

SML (female, academic primary care physician), SAM (female, primary care and palliative care physician), ZNL (female, palliative care physician) and YLC (female, research assistant) analysed the data. SML, SAM, and ZNL have experience with qualitative research and are interested in developing better support systems for end-of-life non-cancer care. YLC attended a qualitative research workshop, but had no prior experience with conducting qualitative research.

### Ethics and sponsorship approval

We obtained ethical approval from the Malaysian Medical Research and Ethics Committee (NMRR ID: NMRR-18-2203-40282), University of Malaya Medical Centre Medical Ethics Committee (20171227-5887), and Hospis Ethics Committee (18/01). The Academic and Clinical Central Office for Research and Development, University of Edinburgh, United Kingdom (AC18003) sponsored the study.

## RESULTS

### Participants

We conducted in-depth interviews with 11 patients (**Table 1**) and 11 physicians (**Table 2**), respiratory care physicians (n = 4), palliative care physicians (n = 4), and primary care physicians (n = 3) [22]. All patients who were approached agreed to participate. They were of different ethnicities, *i.e.* Malay, Chinese, Indian, and Eurasian, and were recruited from primary care clinics, palliative care clinics, respiratory clinics, and wards. Diversity in terms of age and gender was limited, as most patients with severe COPD were older and male. All patients except for one were from the B40 income group, representing the bottom 40% of income earners in Malaysia. There were two HCPs who did not answer the invitation, but of those who did, all consented for recruitment. Of the physicians, six were heads of units and policymakers in their fields.

**Table 1:** Patient participants’ sociodemographic characteristics

Subject	Age band	Sex	Current/most recent occupation	Monthly household income in RM	Years since diagnosis of COPD	Smoking status
PT 01	60–65	Female	Cook	300	30	Former
PT 02	75–80	Male	Engineer	Not known	10	Former
PT 03	70–75	Male	Consultant on water treatment	3000	3	Current
PT 04	65–70	Male	Agriculture work	2600	9	Former
PT 05	80–85	Male	Lorry driver	1000	27	Current
PT 06	65–70	Male	Lorry driver	800	10	Former
PT 07	65–70	Male	Retired military service	7000	8	Former
PT 08	80–85	Male	Mechanic		3	Former
PT 09	70–75	Male	Security	1600	6	Former
PT 10	75–80	Male	Shop owner	3000	5	Former
PT 11	75–80	Male	Retired military service	1000	12	Former

COPD – chronic obstructive pulmonary disease, PT – participant

**Table 2:** Physician participants’ sociodemographic characteristics

Subject	Sex	Specialty	Experience in specialty in years	Place of work	Number of patients seen for COPD palliative care
HCP 01	Male	Internal and palliative medicine physician	20	Hospital	3–5/month
HCP 02	Male	Internal medicine physician	10	Hospital	4/year
HCP 03	Male	Respiratory physician	7	Hospital	
HCP 04	Male	Primary care physician	25	Hospice	10/month
HCP 05	Male	Palliative medicine physician	5	Hospital	4/week
HCP 06	Female	Respiratory physician	10	Hospital	5–10/month
HCP 07	Male	Respiratory physician	15	Hospital	50/year
HCP 08	Male	Palliative medicine physician	14	Hospice	-
HCP 09	Female	Primary care physician	7	Primary care clinic	2/year
HCP 10	Male	Primary care physician	10	Primary care clinic	1/year
HCP 11	Female	Primary care physician	18	Primary care clinic	-

COPD – chronic obstructive pulmonary disease, HCP – healthcare personnel

### Overview of findings

Four main themes emerged. First, there was poor awareness and understanding regarding severe COPD and palliative care. Second, patients were suffering with severe physical, emotional, and psychosocial impacts of the disease. Third, there was lack of accessibility, compassionate, holistic, and coordinated care, except for efforts by a small core group. Finally, cultural issues such as religious norms, spirituality, community, and power hierarchies impacted on patient care and acceptance. We discuss this below, with supporting quotes.

#### Poor awareness and understanding regarding severe COPD and palliative care

There was poor awareness and understanding of COPD, compounded by difficulty in terminology and limited health literacy of the population. There is no Malay equivalent for the term ‘COPD’: patients and physicians often labelled it as ‘asthma’, leading to further misunderstanding and delay in appropriate management.

I don’t know about COPD, I don’t know anything about the full name of COPD, but I know COPD. The ‘asthma COPD’, that’s all I know. *– PT 4, male, 60s.*

Usually, the simplest way is to say ‘asthma due to smoking’. *– HCP 7, respiratory physician.*

Patients also did not fully understand the aetiology of the disease.

He (the traditional healer) said there is something inside the lungs sent by this girl. She keeps attacking. – *PT 07, male, 60s.*

Physicians and patients lacked awareness of the role of palliative care in severe COPD.

Of course, it will be good if there is such service that will allow nurses to go and visit. But I don’t think it is doable by the government? [Interviewer: Actually, we do have such services by an organisation called Hospis Malaysia. Have you heard of it?] No, I never heard of it. – *PT10, male, 70s.*

Cancer was seen to be more important.

Everyone understands lung cancer, lung cancer, ‘ooh cancer’, everyone feels sorry for them. There is a lot of awareness of cancer. There is virtually no awareness of, er, a chronic disease such as COPD. – *HCP 4, primary care physician.*

#### Severe physical, emotional, and psychosocial impact of the disease

The patients described how the disease impacted on every aspect of their lives, causing great physical, social, and emotional suffering to them and their caregivers.

Going anywhere is hard. It’s been over 2 years since I have been downstairs, just staying in this house only. Adoi (cry of pain), Ya Allah (Oh God!). – *PT 01, female, 60s.*

The quality of life experienced by these patients was poor with symptoms of unrelenting breathlessness, pain, and tiredness.

I’m always out of breath, my stomach always hurts, my chest hurts, stomach pains, then I don’t have energy. I‘m weak. No appetite. I lie down for a while then get up, sleep for a while then up again. That’s it (…). *– PT11, male, 70s.*

Some participants felt hopeless of their situation and spoke of their desire to die.

My mood, I don’t want to suffer so much. If cannot be cured, why not let me just die, die already? – *PT 02, male, 70s.*

The disease took away the ability of the patients to work and care for themselves. The financial impact also affected the life of family members and other caregivers.

Most of the COPD patients that I’ve seen are guys, I mean, also happen to be the main breadwinner of the family. When they can’t work, erm, the family tends to have a rougher time. *– HCP 5, palliative care physician.*

#### Lack of accessible, compassionate, holistic, and coordinated care

The response of the Malaysian healthcare system to the needs of patients with severe life-limiting COPD was poor. Patients were unable to access care; they described transport difficulties, crowded, busy clinics without access to oxygen, and no access for disabled people.

(…) one is the parking problems. And the lift (…) I think it is not OKU (those with physical challenges) friendly. *– HCP 10, primary care physician.*

Patients described many incidents when they felt neglected and ignored. There was little communication between patients and physicians and within the healthcare team, leading to uncoordinated care.

[ANONYMISED] hospital never the same doctor. It is difficult. Sometimes the doctor doesn’t know what to do, he looks at you up and down and goes off. That’s all. – *PT 03, male, 70s.*

One of the things I did when I first came back from UK, was I was still using UK, habits of every time I saw a patient I would write to their doctor as to, as to what my discussions were and what was my intervention, so that they would know what I did. After a few years, I realised no one else was doing this. There was zero communication between people dealing with the same cases here. – *HCP 4, primary care physician.*

There were efforts to address these issues by a core group of dedicated health professionals who talked about setting up liaison palliative clinics with respiratory physicians, the inclusion of organ failure into eligibility for palliative services, and the training of doctors. Many of these initiatives were at an early stage, constrained by limitations in resources such as trained personnel, time, and budget.

We have accepted non-cancer patients from day one and after 25 years, the proportion of non-cancer still remains at 10%, despite non-cancer being seen as the single biggest area of need. *– HCP 4, primary care physician working at a hospice.*

One respiratory physician who disagreed with this theme felt that patients were fortunate to get high quality care at low cost. However, he stated that patients with poor prognosis would be discharged to primary care but rarely get referred to palliative care.

#### Cultural issues and power hierarchies impacted on patient care and acceptance

Many participants described how cultural issues such as religious norms and spirituality affected patient care and acceptance of their condition. For example, *redha *(acceptance), an important part of the Islamic religion, was perceived by Muslims to be an expression of their contentment with God’s will. Fate was also important for patients of other faiths: some non-Muslim participants attributed their illness to karma or spiritual imbalance, affecting their approach to healthcare decisions.

As Malay Muslim, I have to accept whatever that comes my way. Everything is given by Allah, our God, so we have to accept it no matter what. That’s all. – *P10 (Muslim), male, 70s.*

From the physicians’ perspective, the acceptance of their condition as fate, led to some patients refusing treatment. This was challenging for HCPs who felt that they could help ease suffering.

They go to the ultimate power. ‘God says I have to suffer. Okay, I am fated to be like this. It’s a test of my will. Alright’ ‘I have some morphine that will override what God wishes (…) you want it?’ (…) ‘No cannot, cannot override God’. It’s, it’s, I find it quite challenging sometimes, where you potentially have erm therapies that may help. But they feel that they don’t want it, that this is a test. – *HCP 4, primary care physician.*

Many of the participants spoke of family involvement in decision making. Patients with COPD were usually elderly, financially unstable and left decisions on end-of-life treatment and finance to their children or other caregivers.

I have always given the responsibility to my daughter to decide what is best for me. My daughter, always talking on my behalf*.* – *PT 08, male, 80s.*

Power dynamics affected the care and the acceptance of the disease. Patients felt that they had lost all control over their lives and were unable to decide on any part of their management due to the perceived absolute power held by physicians.

The doctor does not want to provide me with medication. I'm afraid, they will do something to me, oh, okay this is an old man, let him die. Does not want to provide me with the medication. –* PT 09, male, 70s.*

In many instances, patients gave up and became resigned to their suffering.

I think that with many of our patients with COPD (…) it’s been years since it’s been like that and, and they don’t see anything new, erm (…) so they stop. They stop asking. They stop wanting to do anything else, erm (…) they just sit and suffer. And they don’t ask. *– HCP 4, primary care physician.*

## DISCUSSION

### Statement of principal findings

Our findings illustrate the suffering that people with very severe COPD in Malaysia face at the end of life. This suffering remains hidden from view to all except to the patients and their carers, compounded by a lack of awareness and understanding – or even a name – for COPD and palliative care, both by the public and the healthcare personnel. This results in an inadequate response from the healthcare system and a lack of accessible, coordinated, compassionate and holistic care. There are also social and cultural factors that play important roles in the care and acceptance of the diagnosis in these patients.

### Interpretation in relation to existing literature

Patients in this study showed poor understanding regarding COPD and palliative care. Most came from the B40 income group, highlighting the compounded challenges faced by socioeconomically disadvantaged populations. The poor understanding may be due to poor health literacy, where difficult terminology and complex causes lead to patients and their physicians resorting to the use of the word ‘asthma’ to describe COPD. This confusion in terminology was observed in a study conducted in Malaysia 10 years ago [23]. A study conducted in India showed that only 58% of physicians used the correct terminology for COPD, with a fifth of the patients with COPD identified their disease as being asthma [24]. In contrast, a study conducted in the Netherlands showed that 70% of patients with COPD could name their disease correctly [25]. It may also reflect the challenges of differentiating COPD and asthma in a primary care setting without access to spirometry. A study conducted in Scotland and Colorado showed that of participants who had a spirometry-based diagnosis of COPD made during the study, 52% reported a prior diagnosis of asthma [26].

There was poor understanding of the potential benefits of palliative care in this group of patients with very severe COPD. There appears to be more attention and palliative care resources provided for cancer patients [27]. Some hospices in Malaysia do not accept patients with a diagnosis other than cancer [28]. In Malaysia and many LMICs, chronic respiratory diseases are not considered to be a top priority for research or clinical focus, often being conspicuous by their absence in national reports [29,30]. This lack of prioritisation is likely to be a key component contributing to the suffering of patients with COPD at the end of their life and the poor response of our healthcare system.

COPD affected all aspects of patients’ and their carers’ lives, causing severe physical, emotional and psychosocial suffering. It is ironic that ‘palliative care that aims to provide relief from the symptoms and stress of a serious illness (ideally from a relatively early stage in the disease [31]) was often misunderstood by our participants as ‘giving up on the patient’ [32]. This lack of understanding is not dissimilar to those in other LMICs [33].

### Implications for policy and practice

The Malaysian government has presented a new vision of healthcare transformation where one of the four key priorities is to move from disease-centred care to patient-centred care [34]. However, the physicians in this study had no clear perception of their own role, leading to poor coordination. COPD patients with severe disease frequently seek hospitalisation, creating tension in an already strained healthcare system. A small group of healthcare personnel were trying to effect change and provide palliative care to these patients. However, system change is required to support these efforts. This is similar to challenges in the provision of palliative care for lung diseases in low-resource settings, where this provision is likely to be non-existent or minimal, and further coupled with low access to health services and social and cultural beliefs around palliative care [35].

Future strategies will have to consider the sociocultural issues relevant to the local context. Belief in fate and God may result in negative or positive influences on self-care practices and coping mechanisms, as described by our participants. This agrees with a study looking at self-care practices and support of Malaysian patients with type 2 diabetes [36]. The concept of *redha* (acceptance of fate) among Muslim patients, for example, underscores how religious acceptance can influence health-seeking behaviour, with some patients perceiving medical intervention as conflicting with their spiritual beliefs. This aligns with studies which have shown that religious fatalism can act as both a coping mechanism and a barrier to timely healthcare access [37]. The emphasis on family decision-making and lack of patient involvement in their care reflects traditional Asian cultural values, where collective responsibility often takes precedence over individual autonomy [38]. While this can foster support for the patient, it may also result in delays or conflicts when family members hold differing views on treatment options. Promoting shared decision-making practices may be an option. These findings underscore the need for culturally tailored healthcare practices/interventions that respect patients' beliefs while facilitating informed decision-making to improve the response of the healthcare system to the needs of these patients.

Power hierarchies were also seen to influence patient care, highlighting the role reversal and disempowerment that occur when people present as a patient in the healthcare system. The situation is exacerbated by the higher prevalence of COPD in the lower socioeconomic population [39], leaving them even more at risk of poor-quality healthcare and limited accessibility [40].

A global group of clinicians and representatives of patient support groups have developed a COPD patient charter that comprises six principles of quality care that patients should expect to receive [41]. These include timely access to diagnosis, assessment, and personalised treatment, with maximisation of quality of life without stigma or guilt. These principles directly address the issues highlighted by our study.

Hence, we need systemic change in Malaysia, including provision or enhancing palliative care services where it is non-existent or lacking in healthcare; developing and implementing cultural sensitive targeted education to bridge knowledge gaps among healthcare providers and patients on COPD and palliative care; and the importance of timely referrals to palliative care. In addition to investing in the infrastructure within LMICs, we need to conduct more research to address the inequities for the disadvantaged population, as highlighted by the Global Alliance for Chronic Diseases (Lung Diseases Group) [42].

### Strengths and limitations

Our study offers major new insights into the need for palliative care for chronic non-cancer diseases (specifically, COPD) in a LMIC in Southeast Asia. The participants were representative of patients with severe COPD who are mainly cared for by a small group of specialised HCPs in the public health system. The results of this study cannot be generalised to affluent patients, who are likely to seek care from private healthcare facilities nor patients in different cultural or healthcare settings. Further, Malaysia is a multicultural middle-income country in Asia, so our findings will provide insights to LMICs of similar settings or cultural background. Moreover, the exclusion of participants with impaired cognition or other life-limiting disease may have led to selection biases rendering them applicable to a specific subset of COPD patients but was necessary to allow for data collection. This ensured results were not confounded by other life-limiting conditions (such as cancer) that already attracted palliative care. There was a differing viewpoint by one of the physician participants on the third theme (lack of accessible, compassionate, holistic, and coordinated care). This may indicate that data saturation was not achieved for HCPs. However, there was triangulation between the patient and doctor transcripts, which supported validity of our findings.

## CONCLUSIONS

There needs to be major change within the Malaysian healthcare system to meet the needs of patients with severe, life-limiting COPD. This will be a complex and challenging task, which should start by creating increased awareness of this hidden neglected population amongst communities, physicians, and policymakers. We need to understand the extent of the problem, ensuring an equitable and ethical strategy that can put in place an accessible, coordinated and holistic approach to address the existing gaps highlighted in this paper.

## Additional material


Online Supplementary Document
