# Supplementary material for: Exploring the disease experience and supportive care for people with very severe chronic obstructive pulmonary disease in Malaysia: a multiperspective qualitative study
Source: J Glob Health. 2025 May 23;15:04127. doi: 10.7189/jogh.15.04127 (PMC12100577; doi:10.7189/jogh.15.04127)
Supplement: Online Supplementary Document [file jogh-15-04127-s001.pdf]

Supplement to: Lim ZN, Liew SM, Khoo EM, Pinnock H, McCarthy S, Jeevajothi Nathan J, Pang YK, Hanafi NS, Hussein N, Abu Bakar AI, Chan YL, Sheikh A; RESPIRE Collaboration. Exploring the disease experience and supportive care for people with very severe chronic obstructive pulmonary disease in Malaysia: a multiperspective qualitative study. J Glob Health. 2025;15:04127.

Appendix A: Interview outline for patients

The following outline is designed to be used by the interviewer in a flexible and responsive manner. Patients will be encouraged to define their own experiences and explore their own concepts of end of life care, rather than imposing any already established frameworks. The interviewer will ask a series of open questions and use patient cues to move the interview forward:

Setting the scene

Open questions	Issues that may be explored
**How are you feeling in yourself today?**<br><br>**Can you tell me a little about how your illness started and what has been happening to you since that time?**	Perceived diagnosis: [COPD, emphysema, bronchitis, asthma, breathing problems]<br><br>Other significant conditions<br><br>Key people [family set-up, social and healthcare]<br><br>Main issues and problems that they have recently had or have now [physical, psychological, social, practical, spiritual, information]<br><br>Identify cues to how the patient addresses these problems, and if, and how they approach end of life needs and issues

**What types of help and support are you receiving?**<br><br>**Which people have helped you most?**<br><br>**What is the most helpful thing they do/have done? Are you aware of services that offer support for those who, like you, are facing significant illness? How could you be helped better?**	People/agencies involved and the help they provide [practice, hospital, nursing services, social services, hospice, voluntary organisations, spiritual, informal care, social networks, benefits, aids and equipment, transport, domestic help]<br><br>Awareness of services: who people are, what they can do to help and how they can be accessed<br><br>How services may be improved [access, timeliness, contact, continuity, co-ordination, respect, dignity, privacy, acceptability, communication, information]

Current problems and issues (physical, psychosocial, spiritual, practical) and support available

Open questions	Issues that may be explored
**Have you had any problems with symptoms?**<br><br>**Can you tell me about them?**<br><br>**What tests and treatments are you having?**<br><br>**Do you know what they are for?**<br><br>**Do you have any problems with them?**	Symptomatology [breathlessness, cough, fatigue, pain, confusion, poor appetite, and thirst]<br><br>Investigations [lung function, CXR, blood gases]<br><br>Drug treatment [inhalers, nebulisers, oxygen, tablets]<br><br>Other [pulmonary rehabilitation, chest physiotherapy]<br><br>Complementary therapies<br><br>Effectiveness [do not seem to help, short-lived relief]

	Side-effects, polypharmacy, impact on daily life [time, access, transport, effects on family life/ social life]
**How do you feel in yourself nowadays?**<br><br>**What worries do you have?**	Symptoms of depression and anxiety
**How has your illness affected your social life, relationships with friends and family?**<br><br>**Are there good things about your illness? What has been the most surprising thing about being ill?**	Sense of isolation, support networks available (or not) loss, or gain of friendships<br><br>Explore any benefits [strengthened relationships, loved, cared for]
**Have you thought about why this has happened, the meaning of life...?**<br><br>**Have you thought about what the future holds?**<br><br>**What are the things you most value in life?**<br><br>**What helps you to cope with things? How do you think these are/ will be affected by your illness?**	Life beliefs [Religious faith/cultural background/family traditions/beliefs]<br><br>Previous experience of serious illness (especially COPD) and death in family/friends<br><br>How life beliefs help (or hinder) coping
**What practical help and support are you receiving? How were this help/support arranged?**<br><br>**Are you and *your carer* getting enough help, information and support, or is more needed, and what?**	[social services, benefits, aids and equipment, transport, domestic help]<br><br>Who suggested/helped make the arrangements? Access: [How easy was it to get? How did you find out what was available? How do you feel about asking for support?]

**Have you thought about what kind of help you might want in the future / if your illness fails to get better?**	Concerns: [able to stay at home, alternatives, preferences, fears, concerns about carer's health, (lack of) understanding of future needs]
**Have there been any services/support/people who have been particularly good/helpful, in dealing with symptoms/feelings/spiritual/practical needs?**	Explore successful models of care, and identify any shortcomings, suggestions for improvement, unmet need.

Communication and information needs

Open questions	Issues that may be explored
**What have you been told about your illness, the cause and about treatment and expected progress?**<br><br>**What did you already know?**<br><br>**Are you getting enough facts and information about your illness, the treatments and what the future holds for you?**	Perceived cause of COPD [smoking, family tendency? occupation, pollution, infection....] Attitudes to smoking as a cause of COPD [guilt, defiance, resignation, irrelevant . ..]<br><br>Does this affect how your carers feel about your illness?<br><br>Do you feel this may affect the treatment you get? Do you still smoke?<br><br>Sources of information [friends, family, healthcare professionals,<br><br>leaflets/books/internet/organisations] Appropriateness of information [content, readability, depth of information, understandability Usefulness [helped decision making, reassured, informed choice and consent]

**How do you find talking to your carer about your illness/ feelings/ family affairs?**<br><br>**Can you talk to your carer about how you are really feeling?**	Relationship with carer [able to talk with carer, has illness altered relationship (positive/negative aspects), other close family/friends able to support, concern for carer
**How do you find talking to doctors, nurses and other people?**<br><br>**What do doctors/nurses/other professionals say about the future?**	Opportunities (if any) to discuss the future/end of life preferences [With whom, enough time, able to ask questions/discuss feelings]<br><br>Who makes decisions about care?<br><br>Preferences for care: [discussed? acknowledged? recorded?]
**Do you almost always see the same healthcare professional — or do you see lots of different people?**<br><br>**Have you had recent experience with a hospital discharge, hospital at home, pulmonary rehabilitation, primary/secondary/tertiary care services, out of hours services?**	Relationship with healthcare teams (consultant, Respiratory nurse, GP, palliative care] and effect on<br><br>can: [continuity and co-ordination - knowing you and your situation and likes, knowing the facts, confidence in the one person/team]<br><br>Communication problems between the different services. Effects on care.

Suggestions

Open questions	Issues that may be explored
**What suggestions have you about how to make care better for others like yourself facing your illness in the future?**<br><br>**How could the quality of your life be improved?**	How can services be improved? [Physical, psychosocial, spiritual, practical needs, information services, communication] [for patient and carer]

**What is the one thing that would make life easier for you at present?**	

Appendix B: Interview outline for health care professionals

Introduction and interview strategy

The researcher will explain that she wants to learn about the needs of, and the services used by patients with severe COPD in general. We are interested in the professional carer's perspective concerning the main issues which are affecting *patients* and their informal carer, and how the social and health services are responding, or should respond to their need. The following outline is designed to be used in a flexible and responsive manner.

Setting the scene

Open questions	Issues that may be explored
**Can you tell me about your involvement with *patients with severe COPD's* care?**	**Official role, (and any unofficial duties they may perform for *patients)* and how long they have been taking care of these patients**
**What are the main issues or problems from your point of view in supporting *patients with severe COPD and informal carer'?***	**[COPD, emphysema, bronchitis, asthma, breathing problems etc] any other significant medical problems.**<br><br>***Patients'* illness, symptoms, disabilities, ability to cope etc. *Informal carer's* ability to cope, physical health etc**

**Who is most involved in the care of *patients with severe COPD?***	**Medical, nursing, and social care packages in place. Any palliative care services?**
**Do you believe the needs of *patients with severe COPD/informal carer* are being well met?**<br><br>**How could they be helped better?**	**Recent referrals for more care/support, any services declined/reluctantly accepted by *patients with severe COPD and/or informal carer,* any multi-disciplinary team meetings/ discussions about *patient's* care?**
**What is your view regarding supportive end of life care for patients with severe COPD?**	
**If you think that is appropriate, who do you think should be the main health provider?**	
**What type of services should be offered? Medical, social, psychological, spiritual?**	
**Are there any such services being offered at the current time?**	
**Do you think you have the knowledge and skills to provide these supportive services?**	
**What form of training do you have? And what form of training would be required if you were to provide such services?**	

Current problems and issues (physical, psychosocial, spiritual, practical) and support available

Open questions	Issues that may be explored
Are there any particular symptoms or aspects of treatment that worry/are a problem for you as *patients with severe COPD's* healthcare professional?	Reasons for concern, action taken, solutions proposed / implemented?<br>[main symptoms, Impact on daily life]<br>[recent or planned investigations, drug treatment, pulmonary rehabilitation]]<br>[effectiveness, side-effects, polypharmacy]
How well are patients with severe COPD and informal carer coping?	Concerns about how patients with severe COPD or informal carer are coping and the effect the illness is having on their life. [depression and anxiety, social isolation, support networks available (or not)]
Are there spiritual issues involved in your care for patients with severe COPD and informal carer?<br>Do they talk to you about their values and beliefs?	Awareness (or not) of any important life beliefs for patients with severe COPD and/or informal carer [Religious faith/cultural background/family traditions/beliefs] Any unmet spiritual needs?
Are patients with severe COPD and informal carer getting enough help and support?<br>Do you think that patients with severe COPD will continue to be able to be looked after at home in the future?	What help? [social services, benefits, aids and equipment, transport, domestic help] Who suggested/helped make the arrangements?<br>How easy was it to arrange?<br>Situations/events (physical, psychosocial) that might threaten home care, situations/events that might support continued home care.

What services/support/people/interventions that have been particularly good/helpful, in dealing with the needs of patients with severe COPD or informal carer?	Explore successful models of care, and identify any shortcomings, suggestions for service improvement.

Communication and information needs

Open questions	Issues that may be explored
What information have you received about *patient's* illness, treatment and prognosis?<br><br>Have you given *patients with severe COPD and carer* any information about the illness or support?	Knowledge about COPD, and source of information [formal training, past professional experience, from talking to *patients with severe COPD* or *informal carer,* personal experience, leaflets/books/internet/organizations] Attitudes to smoking as a cause of COPD<br><br>[e.g.blame, irrelevant] Does this affect how you<br><br>feel about caring for *patients with severe COPD* illness?<br><br>Do you, or did you, smoke?<br><br>What information? What support?
How often do *patients with severe COPD* and/or *informal carer* seek your services?<br><br>As part of your professional role do you talk with *patients with severe COPD* about the illness/feelings/domestic circumstance or provide support for *patients with severe COPD* or *informal carer?*	If so, what issues do you discuss? Practical problems, emotional needs, their anxiety, and fears about the future.<br><br>What support is needed: [reassurance, confidence building, safety net, information...]<br><br>Barriers to discussion: [difficulty knowing prognosis, difficult to broach the subject, unwillingness of *patients*

Can you tell me a little about the discussions you have had with *patients with severe COPD* about the prognosis, care available as the COPD progresses, and about any end-of-life preferences?	*with severe COPD/informal carer* to discuss.....]<br>facilitators to discussion: [recent exacerbation, good relationship]<br>Are *patients with severe COPD's* preferences for *his* death/ place of death recorded in the medical records?
Do you think that *patients with severe COPD and their informal carer* are able to communicate well together?	Does this affect your care for them, or their quality of life?
Is the communication between all the different professionals involved in *patients with severe COPD's* care good?<br><br>Is the communication between all the different professionals involved in *patients with severe COPD's* care good?<br><br>Is it easy for you to communicate with them?	Any problems, or improvements, with communication between professionals? [any recent experience with a hospital discharge, hospital at home, pulmonary rehabilitation, primary/secondary/tertiary care services, out of hours services.]

Suggestions

Open questions	Issues that may be explored
Is there anything which would help you to provide care for this patient with severe COPD/ family more effectively?	How can services be improved? [physical, psychosocial, spiritual, practical needs, information services, communication] [for *carer, patients with severe COPD]*
